# Single-tube two-step RPA-CRISPR/Cas12b platform for detection of *Pseudomonas aeruginosa*

**DOI:** 10.1371/journal.pone.0340856

**Published:** 2026-03-18

**Authors:** Haotian Lin, Shichun Wang, Yuanhong Xie, Congyang Cheng, Junhua Jin, Xiaodong Song, Hongxing Zhang

**Affiliations:** 1 Beijing Laboratory of Food Quality and Safety, Beijing Key Laboratory of Agricultural Product Detection and Control of Spoilage Organisms and Pesticide Residue, College of Food Science and Engineering, Beijing University of Agriculture, Beijing, China; 2 Key Laboratory of Dairy Quality Digital Intelligence Monitoring Technology, State Administration for Market Regulation, Inner Mongolia Mengniu Dairy (Group) Co., Ltd., Hohhot, China; Huadong Research Institute for Medicine and Biotechniques, CHINA

## Abstract

*Pseudomonas aeruginosa* is a ubiquitous opportunistic pathogen of significant clinical and public health concern, necessitating the development of rapid and reliable detection methods. Traditional diagnostic approaches, which rely on culture-dependent techniques and biochemical identification, are often labor-intensive, time-consuming, and technically demanding. This study describes a novel single-tube, two-step, rapid detection platform that integrates recombinase polymerase amplification (RPA) with clustered regularly interspaced short palindromic repeats-associated protein Cas12b technology. Through systematic experimental optimization, the study identified an optimal RPA primer pair (F2-R1) and single-guide ribonucleic acid 553 that targets the *lasR* gene of *P. aeruginosa*, with reaction conditions optimized at 42°C and a primer concentration of 10 μM. The RPA-clustered regularly interspaced short palindromic repeats/Cas12b fluorescence detection system (RPA-Cas12b-Fluo) demonstrated a sensitivity threshold of 10 copies of deoxyribonucleic acid (DNA) per reaction and a bacterial detection limit of 50 colony-forming units (CFU) per reaction. When coupled with a lateral flow strip (RPA-Cas12b-LFS), the sensitivity was slightly reduced but remained robust, achieving detection limits of 10² copies and 200 CFU per reaction. Specificity assays confirmed a high discriminatory capacity for *P. aeruginosa* with no cross-reactivity observed against *P. fluorescens*, *P. putida*, or six common foodborne pathogens, thereby validating the specificity profile of the platform. The applicability of the method was further validated by analyzing 20 water samples, which demonstrated 100% concordance with the national standard culture method. These findings have significant implications for improving outbreak surveillance and mitigating the risk of foodborne transmission associated with *P. aeruginosa*.

## Introduction

*Pseudomonas aeruginosa*, a ubiquitous environmental bacterium, is an opportunistic pathogen associated with cutaneous infections, particularly in individuals with burns, scalds, or ocular diseases, potentially leading to sepsis [1]. Chinese national standards GB 8537−2018 and GB 8538−2022 mandate the absence of *P. aeruginosa* in drinking water and specify its detection via membrane filtration, selective culture media, and biochemical tests. However, these traditional methods are laborious, time-consuming, and technically demanding [2]. There is a pressing need for detection technologies that are faster, more accurate, and less costly and technically demanding [3].

Isothermal nucleic acid amplification techniques have emerged as promising tools for the detection of microorganisms in food matrices, exhibiting distinct advantages such as simplicity, rapidity, high efficiency, high sensitivity, broad applicability, and minimal dependency on specialized equipment [4]. Prominent examples of these methods include rolling circle amplification [5], loop-mediated isothermal amplification (LAMP) [6], cross-priming amplification [7], and recombinase polymerase amplification (RPA) [3]. Among these, RPA has gained widespread application in molecular diagnostics, food safety testing, and environmental monitoring due to its operational simplicity and rapidity. Developed by Piepenburg et al. in 2006 and commercialized by TwistDx, RPA enables nucleic acid amplification at a constant temperature of 37–42°C, yielding target products within 30 min [8]. This technique requires minimal instrumentation, unlike other isothermal amplification methods, making it particularly suitable for rapid and low-resource diagnostic applications. Recent studies have expanded the utility of RPA for the detection of *P. aeruginosa*. Jin et al. [9] established an RPA-based method for the rapid detection of *P. aeruginosa*, demonstrating significantly shorter detection times than polymerase chain reaction (PCR) and real-time quantitative PCR, although with reduced sensitivity. Tran et al. [10] developed a multiplex RPA method that achieved high sensitivity without requiring genomic DNA extraction, thereby simplifying the workflow. Xu et al. [11] integrated RPA with magnetic nanobead-based DNA extraction to detect *P. aeruginosa* in clinical samples, such as blood, wound swabs, and sputum, achieving high sensitivity and specificity. Additionally, Yang et al. [12] combined the RPA with LFS to enable visual detection and further enhance its applicability in point-of-care settings. These advancements highlight the potential of RPA as a versatile platform for rapid and accurate microbial detection in diverse applications.

The clustered regularly interspaced short palindromic repeats (CRISPR) and CRISPR-associated protein (Cas) system, first identified in *Escherichia coli* in 1987, has transformed molecular diagnostics since its adaptation as a programmable gene editing tool in 2012 [13]. Current diagnostic platforms primarily use Cas12a (Type V), Cas12b (Type V-B), and Cas13a (Type VI) nucleases, each exhibiting distinct molecular recognition mechanisms [14]. Cas12a demonstrates dual DNA/RNA targeting capacity and mature micro RNA discrimination but requires stringent CRISPR RNA-target complementarity (≥18 nt), shows reduced mismatch tolerance in PAM-distal regions, and is susceptible to nucleic acid secondary structures [14, 15]. In contrast, Cas12b achieves enhanced specificity through minimal single-guide RNA (sgRNA)-target mismatch tolerance (<3 nt) [16], coupled with broad thermotolerance (37–60°C) that enables seamless integration with RPA workflows [17,18]. Its trans-cleavage activity generates multiple DNA fragments per activation event, improving the detection sensitivity [19]. Cas13a leverages collateral RNA cleavage for signal amplification, enabling attomolar-level detection and single-nucleotide polymorphism discrimination [3]. However, nonspecific ribonuclease activity elevates background noise at low target concentrations, whereas CRISPR RNA-target mismatches critically impair cleavage efficiency [13]. These orthogonal CRISPR systems establish multiplexed diagnostic frameworks through PCR/RPA-mediated template amplification coupled with Cas-mediated sequence recognition [20], achieving <10 copies of the detection limit across clinical and environmental matrices [14,19]. The strategic selection of Cas effectors based on the target nucleic acid type, required specificity, and operational constraints enables an optimized assay design for diverse diagnostic applications. Compared with Cas13a, which only cleaves target RNA, and Cas12b, which relies on PCR for template amplification, the RPA-CRISPR/Cas12b detection method amplifies targets via RPA and achieves specific recognition through the Cas12b-sgRNA complex. This method simultaneously activates the trans-cleavage activity of Cas12b to cleave both target double-stranded DNA and nonspecific single-stranded DNA (ssDNA) [21]. It is suitable for quantitative nucleic acid detection in laboratories equipped with specialized instruments and can be adapted for on-site and primary laboratory settings using portable real-time fluorescence detectors or nucleic acid test strips for large-scale sample screening and detection [22].

Integrating the CRISPR-Cas system with RPA has emerged as a promising approach for detecting *P. aeruginosa*. Previous studies explored the combination of RPA with Cas12a and Cas13a. Zhang et al. [23] developed an RPA-CRISPR/Cas12a method targeting the *lasB* gene, using LFS to visualize the results. Yang et al. [24] established a one-tube RPA-CRISPR/Cas12a method for the *lasB* gene, while Liu et al. [25] have demonstrated improved sensitivity using fluorescence detection over agarose gel electrophoresis in an RPA-CRISPR/Cas12a method targeting the *oprI* gene. Zhu et al. [26] have reported a two-step RPA-CRISPR/Cas13a method for the *mexX* gene that showed enhanced sensitivity compared with single-step approaches. Previous studies targeting *P. aeruginosa* have predominantly utilized *lasB*, *oprL*, and *mexX* gene fragments. In contrast, the *lasR* gene-encoding the transcriptional regulator LasR, a central modulator of the *P. aeruginosa* quorum-sensing (QS) system governing virulence and biofilm formation [1], remains unexplored for diagnostic applications. Given its functional significance, evolutionary conservation across *P. aeruginosa* strains, and species-specific sequence features, *lasR* represents a biologically compelling target for detection.

In this study, we developed a novel single-tube, two-step detection platform for *P. aeruginosa* by integrating RPA with the CRISPR/Cas12b technology. The platform was designed to achieve rapid, sensitive, and specific detection of *P. aeruginosa* while minimizing technical complexity and contamination risks. The study objectives included the systematic optimization of primer pairs, sgRNA sequences, and reaction conditions to enhance analytical sensitivity and specificity. Additionally, the specificity of the platform was validated against closely related *Pseudomonas* spp. and common foodborne pathogens to ensure discriminatory accuracy. Practical applicability was evaluated by analyzing unprocessed environmental water samples, demonstrating the platform’s potential for real-world applications.

## Materials and methods

### Materials

The bacterial strains and culture conditions used in this study are listed in Table 1.

**Table 1:** Bacterial strains and culture conditions.

Strains	Source	Culture conditions
*Salmonella enterica*	ATCC 19585	TSB, 37°C, 18 h
*Shigella flexneri*	ATCC 12022	TSB, 37°C, 18 h
*Staphylococcus aureus*	ATCC 25923	TSB, 37°C, 18 h
*Listeria monocytogenes*	ATCC 19111	TSB, 37°C, 18 h
*Escherichia coli*	ATCC 35401	LB, 37°C, 18 h
*Salmonella typhimurium*	ATCC 14028	LB, 37°C, 18 h
*Pseudomonas fluorescens*	ATCC 17556	NA, 30°C, 24 h
*Pseudomonas putida*	ATCC 47054	NA, 30°C, 24 h
*Pseudomonas aeruginosa*	ATCC 9027	LB, 37°C, 24 h

## Methods

### Genomic DNA extraction

The bacterial strains were cultured under the conditions specified in Table 1. Genomic DNA was extracted from 1 mL of bacterial culture using a Bacterial Genomic DNA Extraction Kit (DP302, Tiangen Biotech, Beijing, China) according to the manufacturer’s instructions. The purified DNA was dissolved in 50 μL of nuclease-free water and stored at −20°C for subsequent analysis.

### Construction of standard plasmid

The full-length DNA sequence of the *lasR* gene (720 bp, Gene ID: 881789) from *P. aeruginosa* was synthesized by Tsingke Biotechnology Co., Ltd. (Beijing, China) and cloned into the pUC57 vector. The resulting recombinant plasmid, designated as pUC-lasR, was used as a quantification standard.

### Design and synthesis of primers and probes

RPA primers targeting the *lasR* gene were designed using Primer Premier 5.0. The primer set comprised four forward primers (lasR-F1, lasR-F2, lasR-F3, and lasR-F4) and one reverse primer (lasR-R1) synthesized by Tsingke Biotechnology Co., Ltd. (Beijing, China). Three sgRNA (sgRNA527, sgRNA553, and sgRNA618), an ssDNA probe, and a biotin-labeled probe were designed and synthesized by Synbio Technologies Co. Ltd. (Beijing, China). The nucleotide sequences of all primers, sgRNA, ssDNA, and biotin-labeled probes are provided in Table 2.

**Table 2:** Sequences of primers and probes.

Primers	Sequences(5′-3′)	Target Site (nt)
lasR-F1	CAGCGTGGAAGCGGAAAACCGGGCCGAGGC	390
lasR-F2	GCGGAAAACCGGGCCGAGGCCAACCGTTTC	399
lasR-F3	GGGCCGAGGCCAACCGTTTCATGGAGTCGG	409
lasR-F4	CGGTCCTGCCGACCCTGTGGATGCTCAAGG	437
lasR-R1	ATAATGGCCGCTACGCGGCGGGAGGTCACA	659
sgRNA527	GUCUAAAGGACAGAUUUUCAACGGGUGUGCCAAUGGCCACUUUCCAGGUGGCAAAGCCCGUUGAACUUCAAGCGAAGUGGCACUGACCAGCCGGGAGAAGGAA	527
sgRNA553	GUCUAAAGGACAGAUUUUCAACGGGUGUGCCAAUGGCCACUUUCCAGGUGGCAAAGCCCGUUGAACUUCAAGCGAAGUGGCACCAGUGGUGCGCCAUCGGCAA	553
sgRNA618	GUCUAAAGGACAGAUUUUCAACGGGUGUGCCAAUGGCCACUUUCCAGGUGGCAAAGCCCGUUGAACUUCAAGCGAAGUGGCACGCUUCCGAGCAGUUGCAGAU	618
ssDNA	FAM-TTTTTTT-BHQ1	
Biotin probe	FAM-TTTTTTT-Biotin	

### RPA-CRISPR/Cas12b-Based Fluorescent (RPA-Cas12b-Fluo) Single-Tube Two-Step Method

The RPA-Cas12b-Fluo method was adapted from the protocol established by An et al. [3] and implemented using a SynSor DNA/RNA Isothermal Rapid Amplification Kit (XS-R-101; Synbio Technologies Co., Ltd., Beijing, China) according to the manufacturer’s instructions. The detection workflow was performed in a single tube, comprising two sequential stages: RPA amplification and CRISPR/Cas12b detection.

The RPA amplification mixture was prepared in the reaction tube by thoroughly resuspending the lyophilized isothermal amplification pellet with 12 µL of Amplification Buffer A, followed by sequential addition of 2 µL forward primer (10 µM), 2 µL reverse primer (10 µM), 1 µL magnesium acetate (350 mM), and 1 µL pUC-lasR plasmid. Concurrently, the CRISPR/Cas12b detection mixture was pre-loaded onto the interior surface of the tube cap, comprising 1 µL AaCas12b enzyme (Beijing Synsorbio Co., Ltd. XS-R-001–1), 2.5 µL 10 × AaCas12b Buffer, 1 µL sgRNA (100 ng/µL), 0.5 µL ssDNA reporter (100 µM), and 5 µL ddH₂O. The tube was gently sealed and subjected to RPA amplification at 42°C for 30 min. Following initial amplification, brief centrifugation combined the CRISPR/Cas12b detection mixture with the RPA amplicon. The reaction was immediately transferred to an isothermal amplification instrument (Gene II, OptiGene, UK) for CRISPR/Cas12b detection at 42°C for 60 min, with fluorescence signals monitored in the FAM channel at 1-min intervals throughout the incubation period.

### Optimization of RPA-Cas12b-Fluo Primers

To identify the optimal primer combination for RPA amplification, four forward primers (lasR-F1, lasR-F2, lasR-F3, and lasR-F4) were individually paired with a reverse primer (lasR-R1). Primer concentrations were tested at 1.25, 2.5, 5, and 10 µM to determine the optimal concentration for maximizing amplification efficiency. sgRNA527 was used for CRISPR/Cas12b detection, and fluorescence signals were monitored in the FAM channel at 1-min intervals. Each experiment was repeated three times.

### Optimization of RPA-Cas12b-Fluo sgRNA

Primer pairs lasR-F2 and lasR-R1 were selected for RPA amplification based on prior optimization. Three sgRNA variants (sgRNA527, sgRNA553, and sgRNA618) were individually evaluated for CRISPR/Cas12b detection to determine the optimal sgRNA for target recognition. Fluorescence signals were monitored in the FAM channel at 1-minute intervals to assess the performance of each sgRNA variant. Each experiment was repeated three times.

### Optimization of RPA-Cas12b-Fluo reaction temperature

To determine the optimal reaction temperature for the RPA-Cas12b-Fluo assay, the primer pair lasR-F2 and lasR-R1 was used for RPA amplification, whereas sgRNA527 was used for CRISPR/Cas12b detection. Reaction temperature optimization was performed across a temperature gradient of 37°C, 38°C, 39°C, 40°C, 41°C, and 42°C. Fluorescence signals were monitored in the FAM channel at 1-min intervals to evaluate the amplification efficiency and specificity under each temperature condition. Each experiment was repeated three times.

### RPA-Cas12b-LFS

The RPA-Cas12b-LFS assay was conducted using a SynSor DNA/RNA Isothermal Rapid Amplification Kit (XS-R-101, Synbio Technologies Co., Ltd., Beijing, China). The reaction setup and amplification conditions were identical to those described for the RPA-Cas12b-Fluo method, except that the ssDNA probe was replaced with a biotin-labeled probe. Following amplification, 21.4 µL of ddH_2_O was added to the PCR reaction tube to adjust the total volume to 50 µL, followed by vortex mixing. A Synsor CRISPR detection LFS (XS-R-102; Synbio Technologies Co., Ltd.) was inserted into the reaction tube and incubated at room temperature for 2–5 min. The results were visually interpreted once the liquid front migrated past the control (C) and test (T) lines, with positive signals indicated by the simultaneous appearance of the C and T lines.

### Sensitivity analysis of RPA-CRISPR/Cas12b

To establish a robust fluorescence threshold for distinguishing positive signals from background noise in the RPA-Cas12b-Fluo assay, a sensitivity analysis was performed using negative controls. The fluorescence intensity was measured across eight independent negative control replicates. And the determination of the cut-off threshold was conducted in accordance with the method described by Zhao et al [27].

The sensitivities of the RPA-Cas12b-Fluo and RPA-Cas12b-LFS methods were evaluated using these two approaches.

DNA Sensitivity: Serial dilutions of the pUC-lasR plasmid DNA were prepared at final concentrations of 1.2 × 10^4^, 1.2 × 10^3^, 1.2 × 10^2^, and 1.2 × 10^1^ copies. Each dilution was analyzed using both detection methods to determine the limit of detection (LOD) for plasmid DNA.

P. *aeruginosa* Sensitivity: Genomic DNA was extracted from 1.0 mL *P. aeruginosa* cultures with viable cell counts of 200, 100, 50, and 25 CFU/mL, and was finally eluted in 50 μL of nuclease-free water for preservation at −20°C. The extracted DNA samples were subjected to RPA-Cas12b-Fluo and RPA-Cas12b-LFS analyses to evaluate the LOD of *P. aeruginosa* genomic DNA.

For all sensitivity assays (DNA and bacterial detection), experiments were conducted in biological triplicate (n = 3 independent samples per concentration).

### Specificity analysis of RPA-CRISPR/Cas12b

To evaluate the specificity of the RPA-Cas12b-Fluo and RPA-Cas12b-LFS methods, genomic DNA from a panel of bacterial strains was tested. The bacterial strains used were *Salmonella Enteritidis*, *Shigella flexneri*, *Staphylococcus aureus*, *Listeria monocytogenes*, *E. coli*, *S. typhimurium*, *Pseudomonas fluorescens*, *P. putida*, and *P. aeruginosa*. Each strain was cultured under the conditions specified in Table 1, and genomic DNA was extracted as previously described. The extracted DNA samples were subjected to the RPA-Cas12b-Fluo and RPA-Cas12b-LFS assays to validate the specificity of the detection methods. For all specificity assays, each experiment was repeated three times.

### Water sample detection

Twenty water samples (10 mL each) were processed to evaluate the practical applicability of the RPA-CRISPR/Cas12b assay. Each sample was centrifuged at 8,000 × g for 5 min, and the supernatant was discarded. The pellet was resuspended in 600 μL of phosphate-buffered saline, transferred to a 2-mL tube, and subjected to a second centrifugation at 12,000 rpm for 2 min. The final pellet was retained for genomic DNA extraction using a Bacterial Genomic DNA Extraction Kit. The extracted DNA was analyzed using the national standard microbial culture identification method and RPA-CRISPR/Cas12b assay to validate the accuracy and reliability of the developed platform. Each experiment was repeated three times.

### Statistical analysis

All quantitative experiments were performed with biological replicates (n = 3 independent samples per condition). Statistical analysis was conducted using GraphPad Prism 9.0 (GraphPad Software, USA). For comparisons among multiple groups, one-way analysis of variance (ANOVA) was performed, followed by Tukey’s honestly significant difference (HSD) post-hoc test for pairwise comparisons. In the bar graphs, significant differences between groups are denoted by different lowercase letters (e.g., a, b, c). Specifically, groups labeled with the same letter are not statistically different from each other, whereas groups labeled with different letters are significantly different at *P* < 0.01. Data are presented as the mean ± standard error of the mean (SEM).

## Results

### Screening of RPA-Cas12b-Fluo primers for *P. aeruginosa*

**Fig 1:**
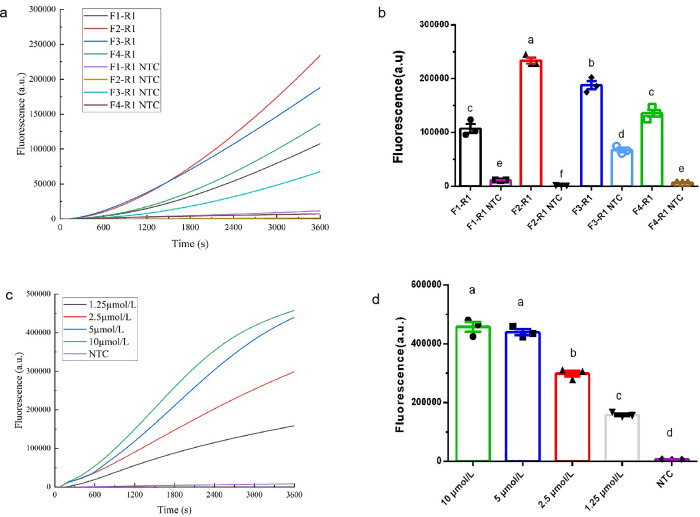
Optimization of RPA-Cas12b-Fluo Primers for *P. aeruginosa* Detection. (a) Amplification kinetics of four primer pairs during 1 h RPA-Cas12b-Fluo reactions; (b) Endpoint fluorescence intensities comparison of primers (1 h); (c) Concentration-dependent amplification profiles of F2-R1 primer pair (1.25-10 μM); (d) Endpoint fluorescence comparison of primer concentrations (1 h). Different lowercase letters above bars indicate statistically significant differences between groups (*P* < 0.01). Data: mean ± SEM (n = 3).

Four primer pairs were synthesized and tested using a *P. aeruginosa* standard plasmid as the template to identify the optimal RPA amplification primers. As shown in Fig 1a, Amplification curves generated during 1 hour of RPA-Cas12b-Fluo analysis indicated that the F2-R1 primer pair exhibited consistently higher fluorescence intensity compared to the other three primer pairs, with no false-positive signals observed. The F2-R1 primer pair exhibited the highest fluorescence intensity (240,000) after 1 h of amplification, while the other three primer pairs showed significantly lower intensities relative to F2-R1 (*P* < 0.01) (Fig 1b). Consequently, the F2-R1 primer pair was selected for subsequent experiments.

To further optimize primer concentration for RPA amplification, the F2-R1 primer pair was tested at concentrations of 1.25, 2.5, 5, and 10 µM. Throughout the 1-hour RPA-Cas12b-Fluo amplification (Fig 1c), the 10 µM F2-R1 primer pair consistently demonstrated higher fluorescence values than other primer concentrations. The 10 µM F2-R1 primer pair yielded a significantly stronger fluorescence signal than the 1.25 µM and 2.5 µM pairs (*P* < 0.01), and performed comparably to the 5 µM pair (Fig 1d). Consequently, a primer concentration of 10 µM was used for RPA amplification.

### Screening of RPA-Cas12b-Fluo sgRNA for *P. aeruginosa* detection

**Fig 2:**
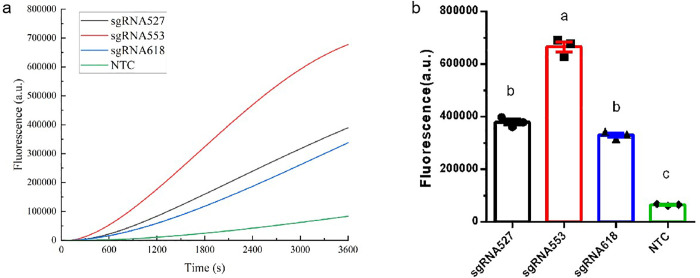
Screening of RPA-Cas12b-Fluo sgRNA. (a) Amplification kinetics of three sgRNA during 1 h RPA-Cas12b-Fluo reactions; (b) Endpoint fluorescence intensities comparison of sgRNA (1 h). Different lowercase letters above bars indicate statistically significant differences between groups (*P* < 0.01). Data: mean ± SEM (n = 3).

To determine the optimal sgRNA for the Cas12b-based detection of *P. aeruginosa*, three sgRNA variants (sgRNA527, sgRNA553, and sgRNA618) were synthesized and evaluated using the *P. aeruginosa* standard plasmid as the template. Fluorescence amplification curves (Fig 2a) demonstrated that during 1 h RPA-Cas12b reactions at 42°C, sgRNA553 exhibited higher fluorescence intensity than sgRNA527 and sgRNA618. Following 1 h amplification, sgRNA553 demonstrated superior performance, generating a significantly higher fluorescence intensity compared to the other sgRNA variants (*P* < 0.01) (Fig 2b). These results indicate that sgRNA553 provides superior target recognition and signal amplification compared with other sgRNA variants. Thus, sgRNA553 was identified as the optimal sgRNA for the Cas12b-based detection of *P. aeruginosa*.

### Optimization of reaction temperature for RPA-Cas12b-Fluo

**Fig 3:**
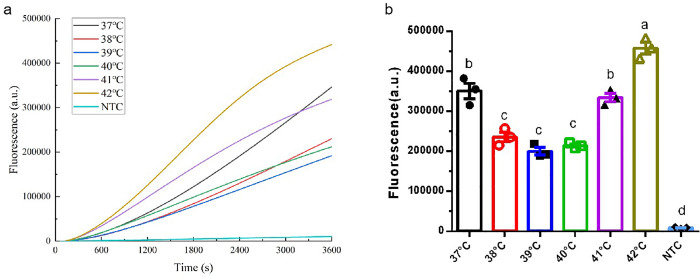
Optimization of the Reaction Temperature for RPA-Cas12b-Fluo. (a) Amplification kinetics of critical temperatures during 1 h RPA-Cas12b-Fluo reactions; (b) Endpoint fluorescence intensities comparison of reaction temperature (1 h). Different lowercase letters above bars indicate statistically significant differences between groups (*P* < 0.01). Data: mean ± SEM (n = 3).

To determine the optimal reaction temperature for the RPA-CRISPR/Cas12b system, a temperature gradient of 37–42°C in 1°C increments was evaluated. Amplification monitoring (Fig 3a) revealed superior fluorescence at 42°C versus other temperatures during RPA-Cas12b-Fluo. Endpoint quantification confirmed this advantage: 42°C samples reached the highest fluorescence intensity, significantly (*P* < 0.01) exceeding other temperature conditions(Fig 3b). These results highlight the temperature-dependent performance of the RPA-CRISPR/Cas12b system, with 42°C providing optimal conditions for both amplification efficiency and signal generation. Based on these findings, all subsequent RPA-CRISPR/Cas12b experiments were conducted at 42°C to ensure maximum analytical sensitivity and specificity.

### Cut-off threshold testing of the RPA-CRISPR/Cas12b-Fluo assay

**Fig 4:**
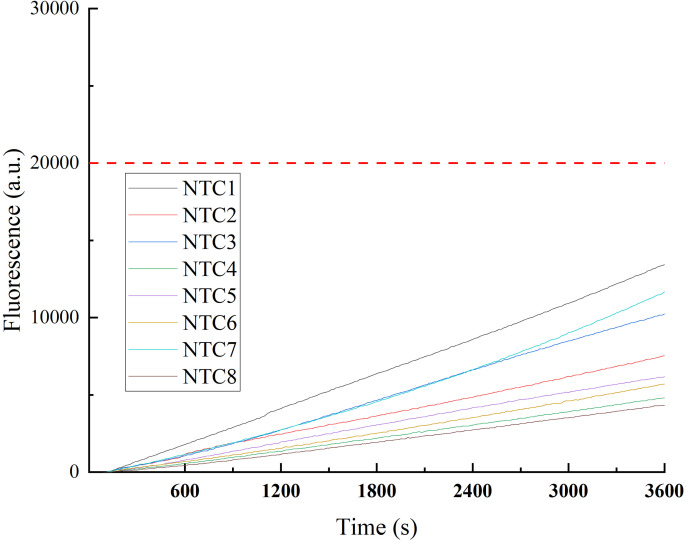
Determination of Fluorescence Threshold for RPA-CRISPR/Cas12b Assay.

Sensitivity testing was performed using negative controls to establish a fluorescence threshold for defining positive reactions in the RPA-CRISPR/Cas12b assay. As shown in Fig 4, eight negative control experiments were conducted, and all fluorescence intensity values remained consistently below 20,000. Based on these findings, a fluorescence intensity value of 20,000 was established as the cut-off threshold for distinguishing positive reactions from background noise. This threshold ensures the reliable discrimination of target-specific signals while minimizing false-positive results.

Fig 4 illustrates the fluorescence intensity values obtained from eight negative control experiments. All values remained below 20,000, establishing a fluorescence intensity of 20,000 as the threshold for distinguishing positive reactions from the background noise.

### Sensitivity analysis of the RPA-CRISPR/Cas12b methods

**Fig 5:**
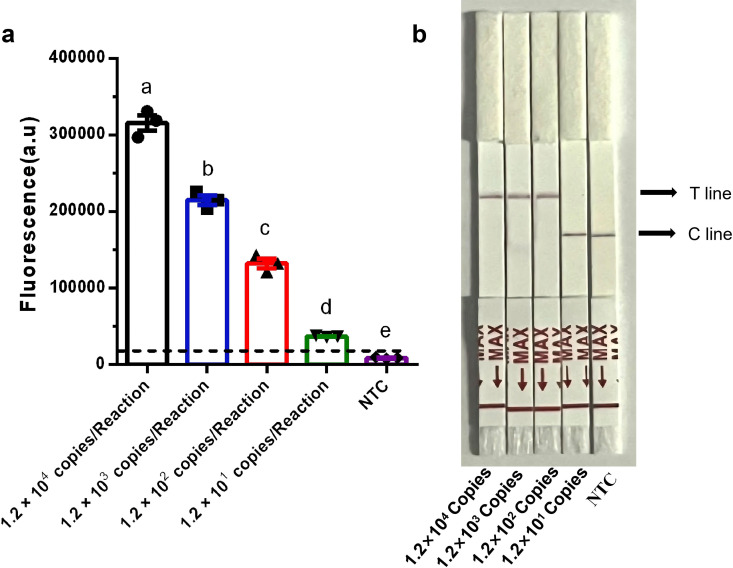
Sensitivity Analysis of RPA-CRISPR/Cas12b Methods on DNA Template. (a) Sensitivity analysis of RPA-Cas12b-Fluo on DNA template. Different lowercase letters above bars indicate statistically significant differences between groups (*P* < 0.01). Data: mean ± SEM (n = 3); (b) Sensitivity analysis of RPA-Cas12b-LFS on DNA template.

Sensitivity for DNA Templates. Using the pUC-lasR plasmid as the template, fluorescence intensities exceeding the established threshold of 20,000 were detected at final concentrations of 1.2 × 10⁴, 1.2 × 10³, 1.2 × 10², and 1.2 × 10¹ copies per reaction. Following 60 minutes of CRISPR/Cas12b-Fluo detection, the mean fluorescence values for 1.2 × 10^4^, 1.2 × 10³, 1.2 × 10², and 1.2 × 10¹ copies per reaction were all significantly exceeding the cut-off threshold of 20,000 (*P* < 0.01) (Fig 5a). Therefore, the LOD for the RPA-Cas12b-Fluo method was determined to be 1.2 × 10¹ copies per reaction. For the RPA-Cas12b-LFS method, a visible positive test band on the LFS was observed when the final concentration of pUC-lasR reached 1.2 × 10² copies per reaction (Fig 5b). These results demonstrate the high sensitivity of both methods, with the RPA-Cas12b-Fluo method achieving a lower LOD than the RPA-Cas12b-LFS method.

**Fig 6:**
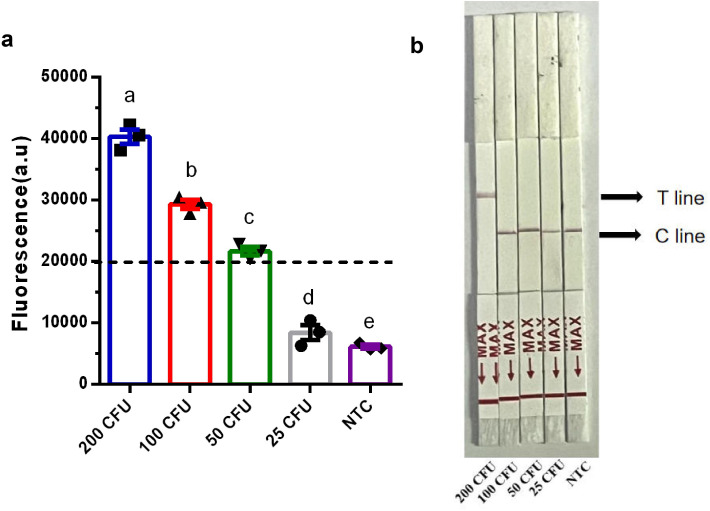
Sensitivity Analysis of RPA-CRISPR/Cas12b Methods on *P. aeruginosa.* (a) Sensitivity analysis of RPA-Cas12b-Fluo on *P. aeruginosa*. Different lowercase letters above bars indicate statistically significant differences between groups (*P* < 0.01). Data: mean ± SEM (n = 3); (b) Sensitivity analysis of RPA-Cas12b-LFS on *P. aeruginosa*.

Sensitivity for *P. aeruginosa* Cells. To evaluate the sensitivity of the RPA-Cas12b-Fluo and RPA-Cas12b-LFS methods for detecting *P. aeruginosa*, genomic DNA was extracted from bacterial cultures with viable counts of 200, 100, 50, and 25 CFU/mL. The extracted DNA was analyzed using both methods. Results indicated that the RPA-Cas12b-Fluo method exhibited fluorescence intensities exceeding the established threshold of 20,000 at bacterial loads as low as 50 CFU/reaction, enabling a clear classification as a positive result (Fig 6a). In contrast, the RPA-Cas12b-LFS method demonstrated an LOD of 200 CFU/reaction with no visible test bands on the LFS at bacterial concentrations below 200 CFU (Fig 6b). These findings highlighted the superior sensitivity of the RPA-Cas12b-Fluo method for detecting low concentrations of *P. aeruginosa*.

### Specificity analysis of the RPA-CRISPR/Cas12b system

**Fig 7:**
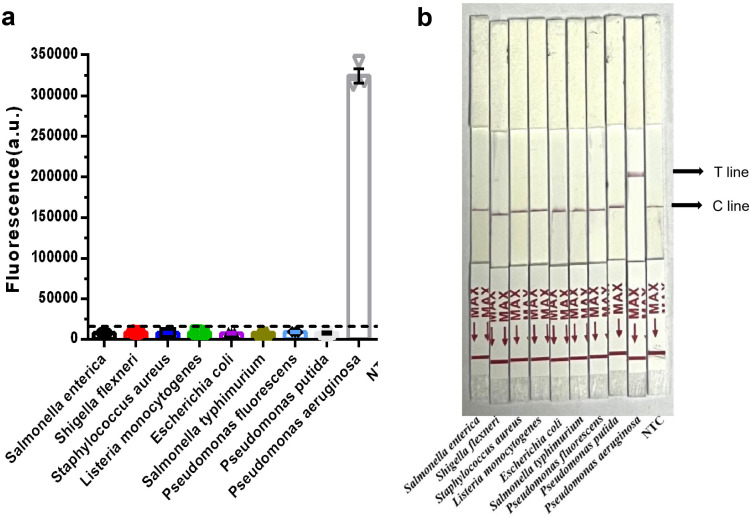
Specificity Analysis of the RPA-CRISPR/Cas12b System. (a) Specificity analysis of RPA-Cas12b-Fluo; (b) Specificity analysis of RPA-Cas12b-LFS.

To evaluate the specificity of the RPA-CRISPR/Cas12b system, genomic DNA from three *Pseudomonas* species (*P. aeruginosa*, *P. fluorescens*, and *P. putida*) and six foodborne pathogens (*Salmonella enteritidis, Salmonella typhimurium, Shigella flexneri, Staphylococcus aureus*, *Listeria monocytogenes*, and *Escherichia coli*) were analyzed. The results are shown in Fig 7. After a 1-h RPA-CRISPR/Cas12b reaction, the RPA-Cas12b-Fluo method generated a fluorescence signal for *P. aeruginosa* that was significantly higher than that of all other tested strains. Non-*P. aeruginosa* strains exhibited no detectable fluorescence signals, and their values were not significantly different from those of the template-free control (Fig 7a). Consistent results were obtained using the RPA-Cas12b-LFS method, in which only *P. aeruginosa* produced a visible test band on the LFS (Fig 7b). These findings demonstrate that the RPA-CRISPR/Cas12b system exhibits high specificity for *P. aeruginosa* with no detectable cross-reactivity against other pathogenic microorganisms, confirming its potential for accurate and reliable detection in complex samples.

### Application of RPA-CRISPR/Cas12b in water sample analysis

To assess the practical utility of the developed RPA-Cas12b-Fluo system for environmental monitoring, commercially available purified water samples were spiked with varying concentrations of *P. aeruginosa* and analyzed using the established assay. As summarized in Table 3, both the conventional microbiological culture-based identification method and the RPA-CRISPR/Cas12b assay identified 15 positive and 5 negative samples, demonstrating complete concordance between the two approaches. These results validated the reliability and accuracy of the RPA-CRISPR/Cas12b system for detecting *P. aeruginosa* in water samples, highlighting its potential as a rapid and effective alternative to traditional culture-based methods for environmental surveillance.

**Table 3:** Comparative Detection of *P. aeruginosa* in Water Samples Using Microbiological Culture and RPA-CRISPR/Cas12b Methods.

Sample ID	S1	S2	S3	S4	S5	S6	S7	S8	S9	S10
Microbiological Culture	+	+	+	+	+	+	+	+	+	+
RPA-Cas12b-Fluo	+	+	+	+	+	+	+	+	+	+
Sample ID	S11	S12	S13	S14	S15	S16	S17	S18	S19	S20
Microbiological Culture	+	+	+	+	+	–	–	–	–	–
RPA-Cas12b-Fluo	+	+	+	+	+	–	–	–	–	–

## Discussion

In recent years, the integration of RPA with the CRISPR/Cas12b technology has emerged as a powerful tool for the rapid detection of various pathogens and antimicrobial resistance genes. This approach has been successfully used to identify *S. enterica* [22], resistance genes such as *mcr-1* and *tet(X4)* [28], and *SARS-CoV-2* [29]. CRISPR/Cas12b has been used to detect diverse pathogenic bacteria, including *Mycobacterium tuberculosis* [30], *Vibrio parahaemolyticus* [31], and *Salmonella spp*. [32]. Despite these advances, few studies have reported the combination of RPA and CRISPR/Cas12b for the rapid detection of *P. aeruginosa*. This study establishes the first RPA-CRISPR/Cas12b platform for detecting *P. aeruginosa*, targeting the previously unexplored *lasR* gene. Unlike existing Cas12a-based methods requiring temperature switching [23,25], our single-tube, two-step workflow maintains a uniform 42°C isothermal condition, eliminating the need for specialized equipment. This design reduces contamination risks and operational complexity, making it ideal for resource-limited settings.

Regarding target gene selection, no studies have reported an RPA-CRISPR-integrated detection method targeting the *lasR* gene of *P. aeruginosa*. Tian et al. [33] developed a graphene oxide-fluorescence-based biosensor for detecting the *lasR* gene in *P. aeruginosa*, while Nair [34] designed CRISPR-Cas9-mediated guide RNAs targeting *lasR* to knock out the gene, thereby reducing biofilm formation and virulence gene expression in *P. aeruginosa*. These studies collectively validate the feasibility of targeting the *lasR* gene for *P. aeruginosa* detection.

In 2023, Liu et al. [25] developed an RPA-CRISPR/Cas12a assay targeting the *oprL* gene of *P. aeruginosa*, achieving a genomic DNA detection sensitivity of 60 fg (approximately eight copies) with an agarose gel electrophoresis detection limit of 6 pg. Similarly, Zhang et al. [23] established an RPA-CRISPR/Cas12a system targeting the *lasB* gene, demonstrating high sensitivity for recombinant plasmid detection, with an LOD of 100 copies/µL via fluorescence analysis and 10¹ copies/reaction using the LFS method. Furthermore, Yang et al. [12] developed a single-tube RPA-CRISPR/Cas12a assay targeting the *lasB* gene of *P. aeruginosa*, achieving a bacterial cell sensitivity of 15.9 CFU/reaction and a 97.62% concordance rate with conventional clinical detection methods. Our platform achieved a sensitivity of 10 DNA copies/reaction (fluorescence) and 50 CFU/reaction, comparable to Cas12a-based assays [23,25] and with enhanced workflow simplicity. While the LFS sensitivity (200 CFU) was lower than Yang et al.’s Cas12a-LFS (15.9 CFU) [12], our method requires no intermediate amplicon transfer, significantly reducing false positives. The *lasR*-targeting system demonstrated 100% specificity against related *Pseudomonas* spp. and foodborne pathogens, outperforming *oprL/lasB*-based assays in discriminatory accuracy [23,25].

Notwithstanding its high analytical sensitivity, this platform exhibits several limitations: restricted matrix diversity currently confined to aqueous samples; uncharacterized susceptibility to matrix interference from complex backgrounds such as food or clinical specimens; and reduced sensitivity in LFS format relative to fluorescence detection. Future validation studies will assess platform performance in clinical matrices while integrating portable readers to enable quantitative LFS analysis. Compared to traditional methods for *P. aeruginosa* detection, our RPA-CRISPR/Cas12b platform offers distinct advantages. Standard culture-based methods, as stipulated in protocols like China’s GB 8537−2018 standard for bottled drinking water, although reliable for determining bacterial viability and serving as the regulatory gold standard, typically require 24–72 hours for completion and involve labor-intensive steps of selective culturing and biochemical confirmation. Molecular techniques like PCR and quantitative PCR (qPCR) offer improved speed and specificity but remain reliant on thermal cyclers and sophisticated laboratory infrastructure, limiting their use in field or point-of-care settings [10]. In contrast, our single-tube, isothermal platform completes detection within approximately 90 minutes, requires only a simple heating block or portable incubator, and reduces the risk of amplicon contamination through its closed-tube, two-step design. The integration with LFS further simplifies result interpretation, making it suitable for non-laboratory environments. However, it is important to note that traditional culture methods retain the unique advantage of providing information on bacterial viability and enabling antimicrobial susceptibility testing, which are crucial in clinical management.

## Conclusion

We successfully developed a single-tube, two-step RPA-CRISPR/Cas12b platform targeting the *lasR* gene of *P. aeruginosa*, which enabled rapid, sensitive, and specific pathogen detection. Through systematic optimization of the reaction conditions, primer pairs, and sgRNA variants, the optimal primer pair (F2-R1) and sgRNA (sgRNA553) were identified. Using fluorescence-based analysis, the platform achieved an LOD of 10 copies/reaction for DNA and 50 CFUs/reaction for bacterial cells. Integration with LFS provides a field-friendly option, achieving an LOD of 10^2^ copies/reaction and 200 CFU/reaction. The assay demonstrated exceptional specificity in distinguishing *P. aeruginosa* from closely related *Pseudomonas* spp. and common foodborne pathogens without cross-reactivity. Validation using 20 water samples showed 100% concordance with the national standard culture methods, confirming its reliability and practical applicability in real-world scenarios. This platform offers a versatile solution for laboratory and field applications owing to its high sensitivity, specificity, and operational simplicity.

## Supporting information

S1 Raw Date Supporting information-Raw Date.
(XLSX)
